# CRISPR-based epigenetic modulation of endothelial dysfunction in sepsis-induced ARDS: a precision medicine framework

**DOI:** 10.1097/MS9.0000000000005221

**Published:** 2026-06-09

**Authors:** Hadiya Majeed, Sabreen Ali, Hafiz Muhammad Haris, Ihsan Qamar

**Affiliations:** a Department of Medicine and Surgery, Azad Jammu Kashmir Medical College, Muzaffarabad, Pakistan; b Department of Medicine and Surgery, King Edward Medical University, Mayo Hospital, Lahore, Pakistan; c Faculty of Medicine, Spinghar University, Kabul, Afghanistan

Sepsis remains a leading precipitant of acute respiratory distress syndrome (ARDS), in which endothelial dysfunction and degradation of the endothelial glycocalyx are established contributors to capillary leak, pulmonary edema, and multiorgan failure^[^1^]^. While advances in lung-protective ventilation and supportive critical care have improved outcomes, therapeutic strategies that directly target endothelial barrier injury remain limited. Parallel advances have separately established the importance of epigenetic regulation in sepsis biology, the development of clustered regularly interspaced short palindromic repeats (CRISPR)-based tools for gene modulation, and the recognition of biological heterogeneity within ARDS; however, these concepts have largely evolved in isolation from one another within critical care research.

Emerging evidence suggests that endothelial injury in sepsis is regulated not only by inflammatory cascades but also by reversible epigenetic mechanisms, including DNA methylation and histone modification^[^2^]^. Building on this foundation, we propose an integrated framework that connects these established domains through three novel considerations. First, rather than broadly targeting inflammatory or immune pathways, we highlight the endothelial glycocalyx as a specific, epigenetically regulated therapeutic focus in sepsis-associated ARDS, supported by emerging mechanistic evidence linking histone modifications to glycocalyx degradation^[^3^]^. Second, recognition of biologically distinct ARDS subphenotypes underscores the heterogeneity of inflammatory and vascular responses among septic patients, suggesting that endothelial injury – and glycocalyx disruption in particular – may not be uniform across individuals and may warrant subtype-specific intervention^[^4^]^. Third, given the rapid evolution of sepsis-induced endothelial dysfunction, we emphasize acute-phase, temporally targeted intervention as a critical determinant of feasibility, positioning epigenetic modulation as an early, biomarker-guided strategy rather than a late rescue therapy.

Within this conceptual framework, CRISPR-based epigenetic editing emerges not as a standalone therapeutic solution but as a tool to enable precise and potentially reversible modulation of endothelial gene expression. Unlike conventional genome editing approaches that permanently alter DNA sequences, catalytically inactive Cas9 (dCas9) fused to transcriptional activators or repressors allows targeted regulation of gene expression without modifying the underlying genome^[^5^]^. In principle, such an approach could be directed toward regulatory regions governing genes involved in glycocalyx synthesis, maintenance, or degradation, informed by endothelial injury biomarkers and ARDS subphenotypes rather than being applied uniformly across all patients.

Importantly, substantial translational challenges remain. Efficient and safe endothelial-targeted delivery systems suitable for acute critical illness are not yet clinically available, and potential off-target effects, immune activation, and regulatory considerations require careful evaluation. Rather than proposing immediate bedside application, we present CRISPR-based endothelial epigenetic modulation as a hypothesis-generating research direction that bridges vascular biology, ARDS subphenotyping, and precision therapeutics. If demonstrated to be feasible and safe in preclinical sepsis models, targeted regulation of endothelial gene expression could ultimately complement existing supportive strategies and shift the paradigm toward early, biologically guided intervention in sepsis-associated ARDS.

## Data Availability

Data will be made available upon reasonable request from the corresponding author.
